# Tremendous bleeding complication after vacuum-assisted sternal closure

**DOI:** 10.1186/1749-8090-6-16

**Published:** 2011-02-09

**Authors:** Arndt H Kiessling, Andreas Lehmann, Frank Isgro, Anton Moritz

**Affiliations:** 1 Department of Thoracic and Cardiovascular Surgery, Johann Wolfgang Goethe University, Frankfurt am Main, Germany; 2 Department of Cardiac Surgery, Klinikum Ludwigshafen, 67069 Ludwigshafen, Germany; 3 Anaesthesiology and Operative Intensive Care, Klinikum Ludwigshafen, 67069 Ludwigshafen, Germany

## Abstract

Vacuum-assisted closure (VAC) of complex infected wounds has recently gained popularity among various surgical specialties. The system is based on the application of negative pressure by controlled suction to the wound surface. The effectiveness of the VAC System on microcirculation and the promotion of granulation tissue proliferation are proved. No contraindications for the use in deep sternal wounds in cardiac surgery are described. In our case report we illustrate a scenario were a patient developed severe bleeding from the ascending aorta by penetration of wire fragments in the vessel. We conclude that all free particles in the sternum have to be removed completely before negative pressure is used.

## Background

Sternal wound healing disorders, with an incidence of 1 to 5%, are a frequent and serious complication following cardio-surgical operations [1].

Vacuum sealing treatment (VST) now represents an established procedure for the care of sternal infections [2] and is used by a majority of cardiac surgical centres in the Federal Republic of Germany for the management of deep, post-sternotomy wounds [1].

Uniform treatment guidelines have not been published thus far. This also applies to contraindications for the use of vacuum sealing treatment in cardiac surgery. Until now, the contraindications have been documented in general surgery and dermatology and focus on the direct effect of suction upon exposed blood vessels [3].

The following case report is intended to point out a possible contraindication from the field of cardiac surgery.

## Case presentation

A 68 year old patient attended our outpatient department in October 2009 to undergo an elective coronary aortic bypass graft (CABG). The patient had triple-vessel coronary artery disease with good systolic left ventricular function (LVEF 58%). Myocardial infarction was not reported. Lately, however, angina complaints occurred at rest. Aside from obesity (BMI 32 kg/m^2^), the risk profile included hyperlipidaemia, non-insulin-dependent diabetes mellitus type II and nicotine abuse of 20 pack/year. Hysterectomy and appendectomy were the only prior operations. The surgical risk was assessed with a EuroSCORE value of 6 and an ASA score of 3. The logistic EuroSCORE calculated a perioperative mortality of 5.8%.

During the uncomplicated elective surgery, the left internal thoracic artery (target vessel was the LAD) and two great saphenous vein segments were anastomosed with the target vessels, marginal branch 1 of the RCX and RCA. (perfusion time 41 min., aortic clamp time 29 min.). The full sternotomy was closed with 3 single wires in the manubrium and 3 figure-of-eight wires in the body of the sternum. The patient was transferred to the intensive care unit intubated and in stable cardiovascular condition. Further progress was uncomplicated. Three-day perioperative antibiotic prophylaxis was carried out with 1.5 g cefuroxime t.i.d. The patient was transferred to an external rehabilitation facility on postoperative day (POD) 12 with a largely un-inflamed wound. The wound margins appeared reddened, but the skin was approximated throughout its length. (Body temperature 37.3°, leucocyte count 8.9/nL)

The patient was readmitted on POD 19 due to wound dehiscence at the junction of the distal and middle thirds. Non-multi-resistant Staphylococcus epidermidis was microbiologically confirmed. The wire cerclages were intact. After preliminary local wound debridement and daily irrigation, surgical wound revision was planned for POD 23 due to increasing putrid secretion. Appropriate antibiotic treatment was started prior to surgery with linezolid 600 mg b.i.d. At operation, the sternum still did not show any dehiscence, the cerclages were sound, and the sternum was not fractured. The wound was managed with bilateral pectoralis muscle flaps, and wound drains were inserted for a period of seven days.

The patient was mobilised on the ward. From POD 40, the skin incision, which was initially closed with interrupted sutures, showed increased erythema, secretion and failing approximation. After removal of the non-absorbable subcutaneous sutures, the medial sternal defect was managed under aseptic conditions with vacuum-assisted suction. The sponge size was about 12 × 7 cm. Suction was set at a continuous -125 mmHg.

At the previously performed debridement, there were loose wires and several rather large bone fragments, which were still held in place with wires. The wires were still intact on the (AP) chest x-ray.

The patient tolerated the treatment well and still remained mobile. Due to her smoking habits, she suffered repeated intense coughing fits while in hospital. As a result, the sponge was changed every three days. Another radiograph was not taken. On POD 51, the patient suffered circulatory depression during the night. The resuscitation team found the patient still responsive in fulminant, hypovolaemic circulatory shock. There were large amounts of blood clots underneath the bulging sternal dressing. Upon removal, there was a spurting haemorrhage in the area of the ascending aorta. Bleeding was stopped by digital compression; the patient was anaesthetised and transferred to surgery under emergency conditions. There were numerous sternal and wire fragments. There was an identifiable bleeding point on the ascending aorta, which was managed with felt sutures. The probable cause of the haemorrhage was penetration by wire fragments during vacuum treatment. The wire fragments had become mobilised, due to the suction effect.

Repeat osteosynthesis and complete debridement of all fragments ensued. The wound was now closed primarily with interrupted sutures. Further vacuum-assisted closure was not used. Linezolid was given without a microbiological confirmation.

The patient was again transferred to the intensive care unit and recovered from the massive transfusion. The ventilation time was 51 hours; she was discharged on POD 72, subjectively well, for further rehabilitation measures.

## Discussion

Sternal wound healing complications are frequently treated by vacuum-assisted closure following surgical debridement [4] This method offers great advantages compared with earlier forms of treating wounds. Better breathing conditions are ensured for the patient by stabilising the two halves of the sternum; granulation is promoted, and the wound environment is improved by aspiration of secretion [5-7].

Vacuum-assisted treatment has, to a large extent, become the standard procedure for treating postoperative wound infections in cardiac surgery [8]. Dehisced sternal wounds are complex, involve major organs and complications can be life-threatening [9].

There are, however, still no authoritative data and studies available on the suction mode and intensity. Similarly, only very few contraindications for the use of vacuum treatment have been published so far. The German Wound Healing Society (DGfW) formulated the following contraindications in 2003 [10]:

▪ coagulation disorders

▪ exposed blood vessels

▪ necrotic wound margin

▪ untreated osteomyelitis

▪ neoplastic wounds

We would like to submit another contraindication, based on the case described above, and draw attention to loose fragments of the sternum and wire. These pieces can be mobilised in the thorax and, as in the reported case, cause a serious complication when it penetrates blood vessels or the ventricles.

## Conclusion

If vacuum sealing treatment is used, close radiological monitoring and examinations are indicated when changing the sponge, and all loose fragments must be removed without compromise.

## Consent

Written informed consent was obtained from the patient for publication of this case report. A copy of the written consent is available for review by the Editor-in-Chief of this journal.

## Declaration of competing interests

The authors declare that they have no competing interests.

## Authors' contributions

AHK drafted the manuscript and was part of the surgical resuscitation team. AL was part of the anaesthesiologist rescue team. FI was part of the surgical team and reviewed the case report. AM reviewed the case report. All authors read and approved the final manuscript.
